# Longitudinal Study of Total and Pathogenic *Vibrio parahaemolyticus* (*tdh*+ and/or *trh*+) in Two Natural Extraction Areas of *Mytilus chilensis* in Southern Chile

**DOI:** 10.3389/fmicb.2021.621737

**Published:** 2021-03-18

**Authors:** Cristina Bacian, Cristobal Verdugo, Katherine García, Josu Perez-Larruscain, Ignacio de Blas, Viviana Cachicas, Carmen Lopez-Joven

**Affiliations:** ^1^Instituto de Medicina Preventiva Veterinaria, Facultad de Ciencias Veterinarias, Universidad Austral de Chile, Valdivia, Chile; ^2^Escuela de Graduados, Facultad de Ciencias Veterinarias, Universidad Austral de Chile, Valdivia, Chile; ^3^Instituto de Ciencias Biomédicas, Facultad de Ciencias de la Salud, Universidad Autónoma de Chile, Santiago, Chile; ^4^Department of Animal Pathology, Instituto Universitario de Investigación Mixto Agroalimentario de Aragón (IA2), Universidad de Zaragoza, Zaragoza, Spain; ^5^Sección Microbiología de Alimentos, Instituto de Salud Pública de Chile, Santiago, Chile

**Keywords:** prevalence, *Vibrio parahaemolyticus*, *tdh*, *trh*, virulence genes, salinity, water temperature, mussels

## Abstract

*Vibrio parahaemolyticus* is the leading cause of seafood-associated bacterial gastroenteritis worldwide. Although different studies have focused on its pattern of variation over time, knowledge about the environmental factors driving the dynamics of this pathogen, within the Chilean territory, is still lacking. This study determined the prevalence of total and pathogenic *V. parahaemolyticus* strains (*tdh* and/or *trh* genes) in mussels (*Mytilus chilensis*) collected from two natural growing areas between 2017 and 2018, using selective agar and PCR analysis. *V. parahaemolyticus* was detected in 45.6% (93/204) of pooled samples from the Valdivia River Estuary. The pathogenic strains carrying the *tdh* and/or *trh* gene were detected in 11.8% (24/204): *tdh* in 9.8% (20/204), *trh* in 0.5% (1/204), and 1.5% (3/204) presented both genes. In Reloncaví Fjord, *V. parahaemolyticus* was detected in 14.4% (30/209) of the samples, pathogenic *V. parahaemolyticus* carrying the *trh* gene was detected in 0.5% (1/209) of the samples, while the *tdh* gene was not detected in the samples from this area. The total count of mauve-purple colonies typical of *V. parahaemolyticus* on CHROMagar was positively associated by multivariate analysis with area, water temperature, and salinity. Similarly, *V. parahaemolyticus* detection rates by PCR had a positive correlation with the area and water temperature. The chances of detecting total *V. parahaemolyticus* in the Valdivia River Estuary are significantly higher than in the Reloncaví Fjord, but inversely, during spring-summer months, the interaction factor between the area and temperature indicated that the chances of detecting *V. parahaemolyticus* are higher in the Reloncaví Fjord. Interestingly, this period coincides with the season when commercial and natural-growing shellfish are harvested. On the other hand, pathogenic *V. parahaemolyticus tdh*+ was significantly correlated with an increase of water temperature. These environmental parameters could be used to trigger a warning on potential hazard, which would influence human health and economic losses in aquaculture systems.

## Introduction

*Vibrio parahaemolyticus* is a Gram-negative halophilic bacterium, ubiquitous in marine and estuarine environments (Joseph et al., 1982; Su and Liu, 2007; Raszl et al., 2016). Gastroenteritis caused by *V. parahaemolyticus* has been reported worldwide. This bacterium is the principal foodborne pathogen associated with the consumption of raw or undercooked seafood in Asia and the United States (Martinez-Urtaza et al., 2004; Su and Liu, 2007; Newton et al., 2012; Wu et al., 2014). In Chile, *V. parahaemolyticus* is considered an important human pathogen, which in 1997 was associated with a large outbreak in Antofagasta, northern Chile, causing more than 300 cases. It was also responsible for several outbreaks between 2004 and 2010 in Puerto Montt, southern Chile, with more than 5,000 cases reported. This region is one of the main shellfish-producing areas, mainly of *Mytilus chilensis* (González-Escalona et al., 2005; Fuenzalida et al., 2006; García et al., 2013).

Particularly, pathogenic *V. parahaemolyticus* isolates capable of inducing gastroenteritis are rare in environmental samples (2–3%) (Nair et al., 2007; Blanco-Abad et al., 2009; Terzi and Martinez-Urtaza, 2016) and are often undetected (Takeda, 1982; Shirai et al., 1990; Honda and Iida, 1993). Furthermore, the thermostable direct hemolysin (*tdh*) and *tdh*-related hemolysin (*trh*) genes are virulence factors commonly associated with pathogenic strains (Broberg et al., 2011; Zhang and Orth, 2013; Raghunath, 2015). However, little is known of their variation pattern over time, as well as environmental factors affecting such variation under Chilean conditions.

The objective of this work is to perform a longitudinal field study to assess temporal and spatial distributions of total *V. parahaemolyticus* and potential pathogenic variants (*tdh*+ and/or *trh*+) associated with *M. chilensis* and, in addition, to address a knowledge gap on the prevalence of *V. parahaemolyticus* strains carrying *tdh* and/or *trh* in natural mussels grown in southern Chile, an area with a strong producer and tourist component. Moreover, this study will also evaluate the association between *V. parahaemolyticus* and environmental parameters, such as temperature and salinity. The information generated by the study will provide new knowledge of the distribution and ecology of *V. parahaemolyticus* across Chile’s southern seawaters. Additionally, the government could use it to manage shellfish harvesting within safe limits and with appropriate post-harvest measures to control and prevent problems for the seafood industry and consumers.

## Materials and Methods

### Sampling Sites and Samples Collection

**FIGURE 1:**
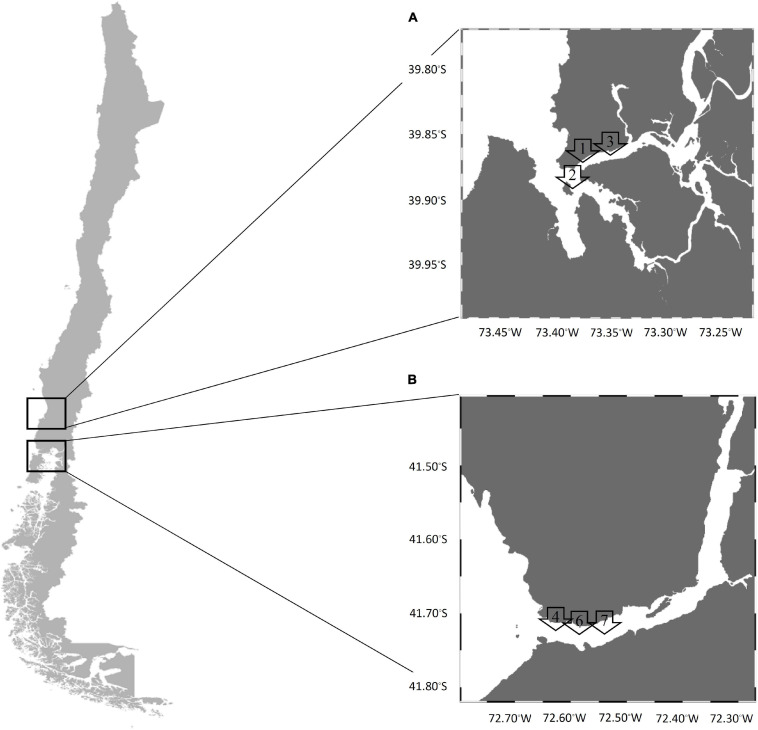
Sampling map in the southern Chile coast **(A)** Valdivia River Estuary and **(B)** Reloncaví Fjord. Arrows denote the sites of each sampling area.

A longitudinal study was realized between January 2017 and May 2018 from two natural extraction areas of mussels (*Mytilus chilensis*) in the southeastern Pacific coast of Chile (Valdivia River Estuary in the Los Ríos Region and the Reloncaví Fjord in the Los Lagos Region) (Figure 1). Briefly, the Valdivia River Estuary is considered a local natural extraction zone of mussels with important tourism as it is near Valdivia city. This area receives the influence of fresh water and has a tidal range less than 2 m where mussels are in the subtidal zone; therefore, they have had little exposure to direct solar radiation, which implies minor internal temperature variations. Monthly, 25 samples were collected at each of three points, point 1 (39°52′33″S, 73°23′08″W), point 2 (39°53′39″S, 73°23′10″W), and point 3 (39°52′10″S, 73°21′19″W).

The Reloncaví Fjord is one of the principal extraction zones of mussels in Chile. This area is near Puerto Montt city and receives the influence of fresh water from the Petrohué and Puelo rivers, and also the influence of ocean water. The tidal range is between 6 and 7 m, and the mussels are found in the intertidal zone exposed to solar radiation for several hours (especially during sunny days, where mussels can reach internal temperatures around 30°C) and adverse environmental conditions with a lower food supply. Monthly, 25 samples were taken at each of three points, point 4 (41°42′33″S, 72°36′60″W), point 6 (41°42′37″S, 72°36′32″W), and point 7 (41°42′37″S, 72°36′06″W).

In addition, environmental parameters, temperature (°C), and salinity water (ppt) were measured each month (YSI Model 30 Salinity, Conductivity and Temperature System, Xylem INC, United States) at all points, at water depths of 0–2 m (sub-surface zone) and 2–8 m (depth zone), in the Valdivia River Estuary and Reloncaví Fjord (Table 1). However, in the present study, the statistical analyses were performed using parameters from subtidal (depth zone) or intertidal zones (sub-surface zone), depending on mussel habitats. Samples were bagged with ice and transported to the laboratory for immediate analysis.

**TABLE 1:** Average temperature and salinity seawater conditions recorded during the different weather stations and for each study areas: Valdivia River Estuary (VRE) and Reloncaví Fjord (RF).

	Seawater temperature (°C)				Seawater salinity (ppt)			
	Sub-surface zone (<2 m)		Depth zone (6–7 m)		Sub-surface zone (<2 m)		Depth zone (6–7 m)	
	VRE	RF	VRE	RF	VRE	RF	VRE	RF
Jan-Feb-Mar 2017	16.5 ± 1.6 (14.8–18.8)	17.3 ± 1.4 (15.4–18.4)	14.7 ± 1.0 (13.3–16.4)	16.0 ± 1.5 (14.0–17.8)	21.8 ± 3.1 (14.5–24.3)	20.3 ± 2.4 (17.7–24.5)	27.2 ± 3.3 (19.3–30.5)	30.6 ± 0.9 (29.2–31.6)
Apr-May-Jun 2017	12.6 ± 2.2 (10.4–16.0)	11.8 ± 1.0 (10.8–13.0)	12.7 ± 1.1 (10.8–14.2)	12.4 ± 0.5 (11.8–13.0)	8.2 ± 5.2 (1.1–15.2)	24.2 ± 3.5 (18.9–26.8)	27.3 ± 6.9 (11.9–32.6)	31.4 ± 0.6 (30.9–32.1)
Jul-Aug-Sep 2017	10.0 ± 0.4 (9.3–10.5)	9.9 ± 0.8 (9.0–11.0)	10.5 ± 0.8 (9.3–11.3)	11.5 ± 0.9 (10.9–13.7)	5.5 ± 5.8 (0.5–16.7)	18.9 ± 4.9 (12.1–30.2)	16.2 ± 13.7 (0.7–30.0)	28.6 ± 5.6 (13.9–31.3)
Oct-Nov-Dec 2017	14.9 ± 1.7 (13.3–16.9)	14.1 ± 3.0 (11.3–17.0)	14.2 ± 1.6 (11.9–17.0)	13.0 ± 1.3 (11.8–14.5)	7.0 ± 4.2 (3.2–12.4)	6.6 ± 1.9 (4.8–8.8)	11.2 ± 9.0 (2.8–27.1)	318 ± 2,7 (29–34,9)
Jan-Feb-Mar 2018	17.2 ± 2.5 (13.6–21.0)	13.6 ± 0.6 (12.8–14.7)	16.0 ± 2.8 (12.6–20.0)	12.9 ± 2.2 (10.2–16.4)	18.0 ± 6.5 (11.5–30.9)	19.0 ± 9.0 (7.6–29.4)	23.2 ± 8.7 (12.3–32.0)	29.0 ± 3.7 (25.3–34.0)
Apr-May 2018	12.0 ± 0.6 (11.4–12.7)	10.0 ± 0.2 (9.8–10.2)	12.6 ± 0.9 (11.1–13.5)	11.9 ± 0.5 (11.4–12.3)	10.2 ± 2.6 (7.0–13.8)	20.9 ± 4.3 (16.0–23.4)	30.0 ± 3.2 (25.3–32.4)	32.7 ± 0.3 (32.5–33.0)

*Each value is the average of 3 months (except average of 2 months April and May 2018), expressed with the mean ± the standard deviation; and the maximum–minimum range below in parentheses. In gray are the conditions that were taken for each study areas [sub-surface in RF and intertidal zone (depth) in VRE].*

### Microbiological Analysis

Samples were tested for numbers of total *V. parahaemolyticus* organisms using specific chromogenic medium for Vibrio growth. Briefly, each mussel was scrubbed with a sterile brush under running tap water to remove any mud. Five pooled samples with five mussels were then formed randomly from each point of the sample, with a total of 15 pooled samples for the area and month. Mussels were opened in sterilized conditions (meat and shell liquid were collected in a sterile laboratory bag) and homogenized (Stomacher^®^ 400 Circulator, Seward Ltd., United Kingdom) at low speed for 1.5 min.

Decimal serial dilutions were prepared in Vibrio phosphate-buffered saline (Vibrio-PBS) composed of 7.650 g NaCl (Merck, Germany), 0.724 g Na_2_HPO_4_ (Merck, Germany), and 0.210 g KH_2_PO_4_ (Merck, Germany) per liter of distilled water, with the pH adjusted to 7.4 using a 1N NaOH solution according to the expected densities of *V. parahaemolyticus* in the sample.

An aliquot of 100 μl of each homogenate pool was streaked onto CHROMagar Vibrio plates (CHROMagar Microbiology, France) and incubated at 37°C for 18–24 h. The temperature of incubation was chosen to enumerate only *Vibrio* capable of growth in human tissues, as the purpose of this study is to determine the relative risks of encountering human pathogens in tissues of consumer food invertebrates. All tests were performed in duplicate. All *Vibrio* colonies and presumptive colonies of *V. parahaemolyticus* (the size and mauve-purple color of the colonies are the main differences with respect to other bacterial genera) were counted manually to determine the density of viable cells (CFU ml^–1^) for each pooled sample (Hara-Kudo et al., 2001; Lopez-Joven et al., 2011). Then, mauve-purple colonies were purified, and each purified isolate was cryopreserved at −80°C for further analysis.

### DNA Extraction

Total DNA was extracted from each purified isolate. Briefly, each isolate was grown onto Tryptic Soy Agar (TSA; Merck, Germany) supplemented with 2% NaCl (Merck, Germany), incubated at 35°C for 18 to 24 h. One colony was then transferred into 10 ml of Tryptic Soy Broth (TSB; Merck, Germany) containing 2% NaCl (Merck, Germany) and incubated under the same conditions. Afterward, for DNA extraction, an aliquot of 1 ml was centrifuged at 4000 × g for 5 min. Then, the supernatant was discarded, and the resultant pellet was resuspended in 500 μl lysis buffer with 5 μl of K proteinase and heated at 56°C for 60 min. The suspension was boiled at 100°C for 10 min and immediately placed on ice for 10 min. The lysate was centrifuged at 4000 × g for 5 min, and the supernatant containing the DNA was transferred to a new tube. Afterward, 500 μl of ethanol at 100% was used for precipitating the DNA. The tube was centrifuged at 16.300 × g for 7 min, the supernatant was discarded, then 200 μl of ethanol at 70% was added, and later it was centrifuged under the same conditions. The supernatant was discarded again, and the tube was dried for at least 1 h at room temperature. Finally, nuclease free water (100 μl) was added, and the tube was heated at 100°C for 3 min. DNA was stored at −20°C until its use as a template for PCR analysis to confirm *V. parahaemolyticus* total [thermolabile hemolysin (*tlh*) gene] and pathogenic organisms carrying the *tdh* and/or *trh* genes.

For positive control, DNeasy^®^ UltraClean^®^ Microbial Kit (QIAGEN, Germany) was used for DNA extraction.

### Molecular Analysis and Virulence Genes Detection

The DNA concentration was measured by spectrophotometry and it was adjusted to 40 ng μl^–1^. *V. parahaemolyticus* primers encoding the *tlh* gene were used to confirm species (Taniguchi et al., 1986), and primers for *tdh* or *trh* were employed to identify pathogenic strains (Bej et al., 1999). Final PCR amplification was performed in a 25 μl volume consisting of 12.5 μl GoTaq^®^ Green Master Mix (Promega, United States), 1.25 μl forward and reverse primers (IDT, United States), 9 μl nuclease free water, and 1 μl of the DNA template.

Positive and negative controls were prepared for each PCR assay. The reference strain ATCC 17802 (carrying *tlh*) and pathogen strain ST64 (carrying *tdh* and *trh*) were used as positive controls and nuclease free water was used as the negative control. The presence of the *tlh*, *tdh*, and *trh* genes was analyzed according to the PCR cycling conditions described by Bej et al. (1999).

Strains without amplification of gene *tlh* by PCR were considered negative for *V. parahaemolyticus* and were not subject to further analysis for *tdh* and *trh* genes. Presence of virulence related genes *tdh* and/or *trh* was determined using independent PCR assays for each gene. The amplification product was separated by electrophoresis in a 1.5% agarose gel.

### Statistical Analysis

A descriptive analysis was used to explore the statistical distribution of colony counts, water temperature (°C), and salinity (ppt) variables. A generalized linear mixed effect modeling (GLMM) approach was used to account for the hierarchical structure of the study design and the correlation present on the data set, due to the longitudinal collection of samples from the same points in each area.

Specifically, two Poisson-based GLMM were used to study the association between environmental factors with the total count of vibrio colonies (model 1) and the total count of mauve-purple colonies in the CHROMagar culture (model 2). Environmental factors (independent variables) such as area, time (sampling month), muscle-weight (gm), water temperature, and salinity were assessed.

Similarly, three logistic-based GLMM were used to assess the relationship between presence/absence of *tlh*, *tdh*, and/or *trh* genes (dependent variables) and the same independent variables mentioned before. Additionally, in all models, the statistical interaction between predictive factors was also carried out. To address the correlation present in the dataset, sampling and month were used as nested random effects. In the case of the logistic-based models, the odd ratios (OR) were computed to estimate the magnitude of the effect of independent variables. The best fitted model was selected based on the computation of the Akaike Information Criteria (AIC), the likelihood ratio test, and the graphical distribution of model residuals. All statistical analyses were performed using the R software version 3.6.1. (R Core Team, 2019), and GLMM was fitted using the lme4 package (Bates et al., 2015) for the R software.

## Results

A total of 1,200 mussels, grouped into 240 pools, were analyzed from three points in the Valdivia River Estuary and 1,045 mussels (209 pools) from three points in the Reloncaví Fjord. Due to severe adverse weather conditions, no samples from the Valdivia River Estuary (October 2017), and neither from the Reloncaví Fjord (June 2017, November 2017, and May 2018) were collected. Additionally, a contaminated pool sample from the Reloncaví Fjord was discarded.

### Environmental Parameters

Collected samples were divided in two areas and three sites per area. Results showed different patterns in temperature and water salinity through the study. Water temperature ranged from 9.3 to 20.0°C in the Valdivia River Estuary in the depth zone and from 9.0 to 18.4°C in the Reloncaví Fjord in the sub-surface zone. In warmer months (January to March) mean temperature was 14.7 ± 1.0°C in 2017 and 16.0 ± 2.8°C in 2018 at the Valdivia River Estuary, while in the Reloncaví Fjord it was 17.3 ± 1.4°C in 2017 and 13.6 ± 0.6°C in 2018 (Table 1). There was no statistical difference in water temperature between sample sites, but statistical differences were found between sampled months.

Water salinity ranged from 0.7 to 32.6 ppt in the Valdivia River Estuary in the depth zone and from 4.8 to 30.2 ppt in the Reloncaví Fjord in sub-surface zone. Water salinity of the Valdivia River Estuary was significantly higher than the Reloncaví Fjord. The highest salinity mean was recorded from April to June in both areas, the Valdivia River Estuary with 27.3 ± 6.9 ppt in 2017 and 30.0 ± 3.2 ppt in 2018, and the Reloncaví Fjord with 24.2 ± 3.5 ppt in 2017 and 20.9 ± 4.3 ppt in 2018. The lowest salinity mean was recorded from October to December 2017, 11.2 ± 9.0 ppt in the Valdivia River Estuary, while a mean of 6.6 ± 1.9 ppt was recorded in the Reloncaví Fjord. Between the sampling point in each area, point 3 in the Valdivia River Estuary was statistically different from the other two points, with a lower salinity. Specifically, this point presented a salinity mean of 2.4 ± 1.5 ppt from August to December 2017.

### Total Vibrios and Presumptive *V. parahaemolyticus* Abundance and Distribution in CHROMagar Vibrio

There was a seasonal distribution of *Vibrio* colonies and presumptive colonies of *V. parahaemolyticus* (indicated by purple-mauve coloration of colonies) incubated at 37°C in CHROMagar Vibrio, to enumerate only Vibrio capable of growth in human tissues. In general, both total vibrios and the presumptive *V. parahaemolyticus* were more abundant in summer than in winter.

**FIGURE 2:**
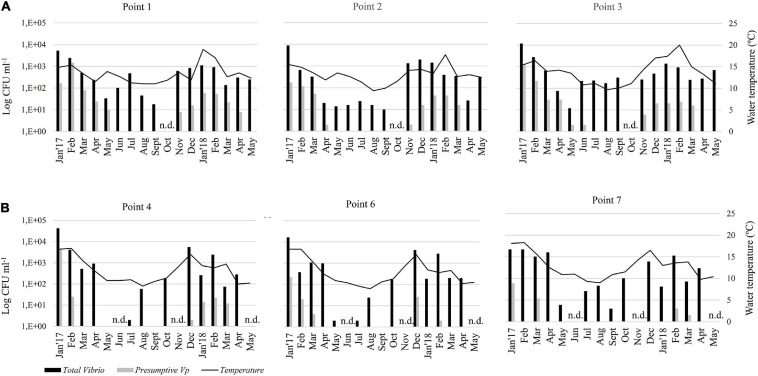
Monthly abundance of total vibrios and presumptive *V. parahaemolyticus* in the pools of mussels’ samples in CHROMagar medium. Histograms show apparent concentrations (UFC ml^–1^) based on CHROMagar plates that represents the arithmetic mean of 5 pools of each point. Samples were collected in two areas: **(A)** Valdivia River Estuary (Points 1, 2, and 3), and **(B)** Reloncaví Fjord (Points 4, 6, and 7). Seawater temperature (°C) is represented by a black line. n.d., not determined.

During the 17 months survey, it was revealed that *Vibrio* species counted in the CHROMagar Vibrio were permanently present in all mussel samples from both areas except, in May 2017 (point 4) and Sept 2017 (points 4 and 6) in the Reloncaví Fjord (Figure 2). The best fitted GLMM for the association between the total count of vibrio colonies in the CHROMagar culture and predictive factors (Table 2) showed a statistical difference in the sites, where the Valdivia River Estuary presented a significant higher total count than the Reloncaví area. When the analysis was stratified by sampling areas, point 1 and point 3 presented a significantly higher total count than other points, indicating statistical differences inside the Valdivia River Estuary (*P* < 0.001), but not in the Reloncaví Fjord points (*P* = 0.191). And the total count of vibrio colonies was statistically associated with the water temperature (Table 2). Conversely, other factors such as total weight, muscle weight, salinity, and the interaction between factors were not associated to this response variable.

**TABLE 2:** Generalized linear mixed effect model results of the association of the total count of vibrio colonies in the CHROMagar culture and predictive factors.

	Estimate	Standard error	*P*-value
Intercept	−0.24	0.21	
**Area**			
-RF	Ref		
-VRE	0.20	0.06	<0.001
Water temperature (°C)	0.08	0.01	<0.001

*RF, Reloncaví Fjord; VRE, Valdivia River Estuary.*

On the other hand, the 17-months survey revealed that presumptive *V. parahaemolyticus* in CHROMagar Vibrio were absent in almost all mussel samples from June to Sept 2017, and April and May 2018 in the Valdivia River Estuary, and from April to Oct 2017 and also April and May 2018 in the Reloncaví Fjord (Figure 2). The best fitted GLMM for the association between the total count of mauve-purple colonies in the CHROMagar culture and predictive factors (Table 3) showed a similar pattern than the total count of the vibrio colonies model. The Valdivia River Estuary area presented a significantly higher count than the Reloncaví Fjord, and water temperature was positively associated with this response variable. However, salinity was negatively associated with the count of mauve-purple colonies. In addition, when a stratified analysis of the presumptive *V. parahaemolyticus* by area was performed, statistical differences were observed between points (*P* < 0.001), where in the Valdivia River Estuary point 1 presented a significantly higher presumptive *V. parahaemolyticus* count than points 2 and 3. Points 6 and 7 in the Reloncaví Fjord had a significantly lower presumptive *V. parahaemolyticus* count than point 4.

**TABLE 3:** Generalized linear mixed effect model results of the association of the total count of mauve-purple colonies in the CHROMagar culture and predictive factors.

	Estimate	Standard error	*P*-value
Intercept	−11.72	1.95	
**Area**			
-RF	Ref		
-VRE	12.38	2.10	<0.001
Water temperature (°C)	0.70	0.12	<0.001
Salinity	−0.02	0.01	0.03
Area x water temperature			
-RF x °C	Ref		
-VRE x °C	−0.69	0.13	<0.001

*RF, Reloncaví Fjord; VRE, Valdivia River Estuary.*

Additionally, the interaction between area and temperature was significant, showing that during warm months the area effect is reversed, because the Valdivia River Estuary presented lower counts than the Reloncaví Fjord. This means that a higher count of mauve-purple colonies is expected in the Valdivia River Estuary over the year, but during the summer the risk of finding mauve-purple colonies in mussels increases significantly in the Reloncaví Fjord area. Other factors and interactions assessed were not significant.

### Prevalence of Total and Potentially Pathogenic *V. parahaemolyticus* Abundance and Distribution

**FIGURE 3:**
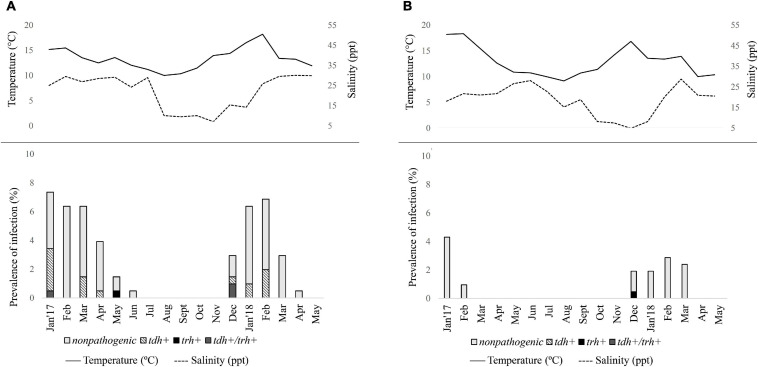
Seasonal distribution of total and pathogenic *Vibrio parahaemolyticus* in *Mytilus chilensis* from January 2017 to May 2018 in **(A)** Valdivia River Estuary and **(B)** Reloncaví Fjord of southern Chile. Each line or bar represents the arithmetic mean of three points. Solid line represents seawater temperature (°C); dashed line represents salinity (ppt) during each campaign.

A total of 204 pools (from 240 pools grouped to presumptive culture) from the Valdivia River Estuary and 209 from the Reloncaví Fjord (all presumptive culture pools) were assessed. The prevalence of total *V. parahaemolyticus* by targeting the *tlh* gene was 45.6% in the Valdivia River Estuary (93/204 pools) and 14.4% in the Reloncaví Fjord (30/209 pooled samples) (Table 4). There were statistical differences between the sampled sites (*P* < 0.001), but not between the sampled points within each area. A seasonal distribution of *V. parahaemolyticus* was observed over time, increasing in prevalence during warm months, and decreasing in cold months. Total *V. parahaemolyticus* was detected from January to June 2017 and from December 2017 to April 2018 in the Valdivia River Estuary (Figure 3A). On the other hand, a lower prevalence was observed in the Reloncaví Fjord, with total *V. parahaemolyticus* detected from January to February 2017 and from December 2017 to March 2018 (Figure 3B).

**TABLE 4:** Prevalence of total *V. parahaemolyticus* (*tlh*+) and potential pathogenic *V. parahaemolyticus* (*tdh*+ and/or *trh*+) in pooled samples of *M. chilensis* from Valdivia River Estuary and Reloncaví Fjord stratified by weather season (summer, fall, winter and spring).

	Valdivia River Estuary					Reloncaví Fjord				
	Pooled samples	Total *Vp*	Potential pathogenic *Vp*			Pooled samples	Total *Vp*	Potential pathogenic *Vp*		
					
		*tlh*+	*tdh*+	*trh*+	*tdh*+*/trh*+		*tlh*+	*tdh*+	*trh*+	*tdh*+*/trh*+
	*n*	*n* (%)	*n* (%)	*n* (%)	n (%)	*n*	*n* (%)	*n* (%)	*n* (%)	*n* (%)
Jan-Feb-March 2017 (Summer)	44	41 (20.1%)	10 (4.9%)	0	1 (0.5%)	44	11 (5.3%)	0	0	0
Apr-May-Jun 2017 (Fall)	42	12 (5.9%)	1 (0.5%)	1 (0.5%)	0	30	0	0	0	0
Jul-Aug-Sept 2017 (Winter)	39	0	0	0	0	45	0	0	0	0
Oct-Nov-Dec 2017 (Spring)	19	6 (2.9%)	3 (1.5%)	0	2 (1.0%)	30	4 (1.9%)	0	1 (0.5%)	0
Jan-Feb-March 2018 (Summer)	37	33 (16.2%)	6 (2.9%)	0	0	45	15 (7.2%)	0	0	0
Apr-May 2018 (Fall)	23	1 (0.5%)	0	0	0	15	0	0	0	0
**Total**	**204**	**93 (45.6%)**	**20 (9.8%)**	**1 (0.5%)**	**3 (1.5%)**	**209**	**30 (14.4%)**	**0**	**1 (0.5%)**	**0**

*Seawater conditions were intertidal zone (6–7 m) and sub-surface zone (<2 m) for Valdivia River Estuary and Reloncaví Fjord, respectively. Some deviations in the size of the pooled samples from the surveillance scheme originated due to environmental conditions in some months that made surveillance not possible and also the absence of mauve-purple colonies in CHROMagar Vibrio or no isolation of them.*

Significant differences were observed in the presence of pathogenic variants between areas (*P* < 0.001). Prevalence of pathogenic *V. parahaemolyticus* (*tdh*+ and/or *trh*+) was 11.8% (24/204) in the Valdivia River Estuary, with the presence of the *tdh* gene in 9.8% (20/204) of samples, while the *trh* gene was present in 0.5% (1/204) of samples, and 1.5% (3/204) presented both genes (*tdh*/*trh*) (Table 4 and Figure 3A). In the case of the Reloncaví Fjord, prevalence of pathogenic *V. parahaemolyticus* (*tdh*+ and/or *trh*+) was 0.5% (1/209 pools), with the detection of only the *trh* gene (1/209) in December 2017 (Table 4 and Figure 3B).

### Multivariate Analysis to Determine the Influence of Environmental Factors in the Presence of *V. parahaemolyticus*

A GLMM was used to determine the presence or absence of total *V. parahaemolyticus* (*tlh* gene). The best fitted model included area, water temperature, salinity, and pool sample weight (without shell), and also the interaction between area and water temperature. In the case of salinity and muscle-weight, borderline significance was observed (*P* < 0.10), while the rest of the fixed effect parameters showed *P*-values lower than 0.001 (Table 5).

**TABLE 5:** Generalized linear mixed effects model results of the association between the presence of total *V. parahaemolyticus* and predictive factors.

	Estimate	Standard error	*P*-value	OR mean	95% CI	
					Lower	Upper
Intercept	−20.9	2.7	< 0.001			
**Area**						
-RF	Ref					
-VRE	5.8	0.9	< 0.001	332.6	60.7	1821.9
Water temperature (°C)	1.0	0.1	< 0.001	2.6	2.1	3.4
Water salinity	0.0	0.0	0.069	1.0	1.0	1.1
Muscle weight	0.0	0.0	0.099	1.0	1.0	1.1
**Area x water temperature**						
-RF x °C	Ref					
-VRE x °C	−0.4	0.1	< 0.001	0.6	0.6	0.8

*RF, Reloncaví Fjord; VRE, Valdivia River Estuary; Muscle weight, pool sample weight (without shell); OR, Odds ratio.*

The presence of *V. parahaemolyticus* (*tlh*+) was positively associated with the area and water temperature, where the OR value estimated that the chances of finding *V. parahaemolyticus* in the Valdivia River Estuary were 332.6 times higher than the Reloncaví Fjord, additionally with every increase in the temperature unit (°C), the chances of detecting *V. parahaemolyticus* raised 2.6 times.

Conversely, the interaction factor between area and water temperature had a negative slope, with an OR equal to 0.60, which indicated that during the warming months the chances of finding *V. parahaemolyticus* were higher in the Reloncaví Fjord than in the Valdivia River Estuary. This pattern suggests that the latter area presents a higher endemic prevalence than the Reloncaví Fjord. However, the presence of *V. parahaemolyticus* in the Reloncaví Fjord is more sensitive to changes in temperature.

Although salinity and muscle-weight were also positively associated with the presence of *V. parahaemolyticus*, the evidence supporting this association is not as robust as with the other predictors (Table 5). Additionally, a GLMM was used to determinate the presence or absence of pathogenic *V*. *parahaemolyticus* with independent variables such as area, time (sampling month), muscle-weight (gm), water temperature, and salinity. However, the presence of pathogenic *V. parahaemolyticus* (*tdh* gene) was statistically associated only with the water temperature, where the chances of detecting the *tdh* gene increased 1.56 times for each degree increase in water temperature (*P* < 0.001). On the other hand, no predictor was associated with the presence of the *trh* gene.

## Discussion

The present study reports on the environmental dynamics of *Vibrio* species in two natural extraction areas used for mussel harvesting in the southeastern Pacific coast of Chile. Parameters influencing their ecology were identified by monitoring total vibrios and potential pathogenic *V. parahaemolyticus* for 17 months (2017–2018).

Overall, the annual dynamics of presumptive *V. parahaemolyticus* (mauve-purple colonies) correlated with temperature and salinity, in contrast with recent findings by Vezzulli et al. (2015) and Lopez-Joven et al. (2018) in Mediterranean lagoons.

*Vibrio parahaemolyticus* has been the object of laboratory surveillance in Chile, since 2000, because of the outbreaks of foodborne diseases that have occurred in the past years (Heitmann et al., 2005; García et al., 2009; Raszl et al., 2016). However, regulatory limits are not established for *V. parahaemolyticus* in the internal consumption of seafood in Chile (Raszl et al., 2016).

Numbers of total *V. parahaemolyticus* organisms increased when seawater temperatures were high (Kaneko and Colwell, 1973; DePaola et al., 1990; Duan and Su, 2005; Parveen et al., 2008; Cruz et al., 2015). A seasonal trend of increasing numbers of *V. parahaemolyticus* was observed when seawater temperatures increased. The outbreak of foodborne diseases in 2004 and 2005 in Puerto Montt, in the Los Lagos Region, was related to the increase of water temperatures above 16°C in the summer months (González-Escalona et al., 2005; Fuenzalida et al., 2006; García et al., 2013).

Growth rates of pathogenic *Vibrio* strains (*tdh*-positive) may be higher than growth rates of confirmed *V. parahaemolyticus* (*tlh*-positive), and therefore we may be underestimating *tlh*-positive cells due to the high incubation temperature (Alarcón Elvira et al., 2020). However, in this study, the incubation temperature to enumerate culturable *Vibrio* was chosen to select for human pathogens that would optimally grow at 37°C. Surprisingly, the present study showed that during warm months there are increased chances of finding *V. parahaemolyticus* in the Reloncaví Fjord than in Valdivia River Estuary, supporting the fact that the temperature was an important factor associated with total *V. parahaemolyticus*. In fact, the two areas have different characteristics that may affect the prevalence of *V. parahaemolyticus*. Mussels in the Reloncaví Fjord were in the intertidal zone, exposed to solar radiation for several hours (especially during sunny days), and adverse environmental conditions with a lower food supply. During summer, exposed mussels can reach internal temperatures around 30°C which largely favors the *V. parahaemolyticus* growth (García et al., 2013). On the other hand, surface seawater temperatures are higher in the Valdivia River Estuary (Garcés-Vargas et al., 2013), and mussels are in the subtidal zone thus spending more time underwater to filter bacteria, which favor an endemic prevalence compared with the Reloncaví Fjord. Additionally, subtidal located mussels are less exposed to direct solar radiation, which implies minor internal temperature variations.

To our knowledge the Valdivia River Estuary does not have a prevalence of *V. parahaemolyticus* in mussels. Prevalence of *V. parahaemolyticus* carrying the *tdh* gene in the Valdivia River Estuary agrees with other worldwide studies; DePaola et al. (2003) found that 13% of oysters carry the *tdh* gene, Duan and Su (2005) found samples with less than 10%, and Lopez-Joven et al. (2015) found 6.7%. It was possible to detect strains of *V. parahaemolyticus* with both genes (*tdh* and *trh*) in environmental samples.

The prevalence of potential pathogenic *V. parahaemolyticus* (*tdh* and/or *trh*) in the Valdivia River Estuary is higher than in the Reloncaví Fjord, and these results coincide with those obtained by Aranda et al. (2015), who did not detect pathogenic *V. parahaemolyticus* in mussels from the Reloncaví Fjord. We show that the presence of pathogenic *V. parahaemolyticus* carrying the *tdh* gene was statistically associated with the water temperature, since the probability to detect the *tdh* gene increased 1.56 times for each increase in this factor.

However, when the interaction factor between area/time and consequently area/temperature was considered, it was suggested that during the warming months the chances of finding *V. parahaemolyticus* are higher in the Reloncaví Fjord than in the Valdivia River Estuary, locating Reloncaví Fjord as a high-risk zone of *V. parahaemolyticus.* Although surface seawater temperatures are higher in the Valdivia River Estuary, it seems that differences are not enough on their own to favor the growth of pathogenic variants, and consequently, the risk could be the same in both locations. This interaction could explain the outbreaks in Puerto Montt, in the Los Lagos Region related to the consumptions of uncooked or raw bivalves, with more than 1,500 and 3,725 cases reported in 2004 and 2005, respectively, both in warm months (González-Escalona et al., 2005; Fuenzalida et al., 2006; García et al., 2013).

The influence of water salinity as an environmental parameter is not as well defined as water temperature. Different studies have report negative, but also positive correlations between salinity and the abundance of potential *V. parahaemolyticus*. A negative correlation was reported by Lopez-Joven et al. (2015) and Davis et al. (2017). Moreover, a positive correlation was detected by DePaola et al. (2003), Zimmerman et al. (2007), and Martinez-Urtaza et al. (2008). Although water salinity and muscle-weight were positively associated with the presence of *V. parahaemolyticus* (*tlh* gene) in our study, it was not statistically associated (P > 0.05) with these parameters. However, in the case of the weight of the meat (without shell), it is necessary to consider that larger mussels have a greater chance of harboring total bacteria, but not pathogenic bacteria, which would indicate that the presence of pathogenic bacteria is something typical of the environment and not of the mollusk.

Nonetheless, several other factors can influence the presence, abundance, and distribution of total and pathogenic *V. parahaemolyticus* in mussels, such as plankton composition (Turner et al., 2013), dissolve oxygen (Parveen et al., 2008; Davis et al., 2017), particulate organic matter availability (Thickman and Gobler, 2017), and chlorophyll (Zimmerman et al., 2007; Parveen et al., 2008). More studies considering these environmental factors and its interactions must be done.

## Conclusion

In conclusion, these results demonstrate that the prevalence of total and pathogenic *V. parahaemolyticus* have a seasonal distribution over time, increasing in warm months and decreasing in cold months, in both areas of southern Chile. This fluctuation can be explained by the positive association with water temperature. Additionally, pathogenic *V. parahaemolyticus* strains with *tdh* or *trh* and both genes (*tdh* and *trh*) were detected in environmental samples. Considering that the Los Lagos Region is one of the main bivalve extraction areas, postharvest handling temperature should be considered, as inadequate temperatures can increase *V. parahaemolyticus* abundance and potential pathogenic *V. parahaemolyticus* (*tdh* and/or *trh*) in bivalves. These results provide information about the distribution of *V. parahaemolyticus* and could help to create measures to control future problems related to shellfish harvesting and consumption in the country.

## Data Availability Statement

The raw data supporting the conclusions of this article will be made available by the authors, without undue reservation.

## Ethics Statement

The animal study was reviewed and approved by the Cómite de Bioética “Uso de animales en la investigación”—Universidad Austral de Chile.

## Author Contributions

CL-J conceived the study idea. CL-J, CV, KG, JP-L, and VC designed the sampling collections and experimental procedures. CB and JP-L performed the microbiology and molecular analyses. CL-J, CB, CV, and IB performed the statistical analysis and analyzed the data. All authors wrote, discussed, and approved the final version of this manuscript.

## Conflict of Interest

The authors declare that the research was conducted in the absence of any commercial or financial relationships that could be construed as a potential conflict of interest.
